# Characteristics of US Health Care Providers Who Counsel Adolescents on Sports and Energy Drink Consumption

**DOI:** 10.1155/2014/987082

**Published:** 2014-03-24

**Authors:** Nan Xiang, Holly Wethington, Stephen Onufrak, Brook Belay

**Affiliations:** ^1^University of Michigan Medical School, Ann Arbor, MI 48109, USA; ^2^The CDC Experience Applied Epidemiology Fellowship, Scientific Education and Professional Development Program Office, Centers for Disease Control and Prevention, Atlanta, GA 30341, USA; ^3^Division of Nutrition, Physical Activity, and Obesity, National Center for Chronic Disease Prevention and Health Promotion, Centers for Disease Control and Prevention, 4770 Buford Hwy NE, MSK-26, Atlanta, GA 30341, USA

## Abstract

*Objective*. To examine the proportion of health care providers who counsel adolescent patients on sports and energy drink (SED) consumption and the association with provider characteristics. *Methods*. This is a cross-sectional analysis of a survey of providers who see patients ≤17 years old. The proportion providing regular counseling on sports drinks (SDs), energy drinks (EDs), or both was assessed. Chi-square analyses examined differences in counseling based on provider characteristics. Multivariate logistic regression calculated adjusted odds ratios (aOR) for characteristics independently associated with SED counseling. *Results*. Overall, 34% of health care providers regularly counseled on both SEDs, with 41% regularly counseling on SDs and 55% regularly counseling on EDs. On adjusted modeling regular SED counseling was associated with the female sex (aOR: 1.44 [95% CI: 1.07–1.93]), high fruit/vegetable intake (aOR: 2.05 [95% CI: 1.54–2.73]), family/general practitioners (aOR: 0.58 [95% CI: 0.41–0.82]) and internists (aOR: 0.37 [95% CI: 0.20–0.70]) versus pediatricians, and group versus individual practices (aOR: 0.59 [95% CI: 0.42–0.84]). Modeling for SD- and ED-specific counseling found similar associations with provider characteristics. *Conclusion*. The prevalence of regular SED counseling is low overall and varies. Provider education on the significance of SED counseling and consumption is important.

## 1. Introduction

Sugar-sweetened beverages (SSB) are drinks sweetened with various forms of sugars that add calories and include, but are not limited to, soda, fruit ades and fruit drinks, and sports (SD) and energy drinks (ED). On average, SSB intake contributes approximately 300 kilocalories to the daily intake of adolescents 12–19 years old in the United States [1]. Regular consumption of these caloric drinks can increase the risk for obesity [2] and dental caries [3]. In particular, sports and energy drinks (SEDs) are relatively new products that are increasingly marketed to adolescents [4]. Furthermore, purchase and consumption of these drinks by adolescents appear to be common [1, 5, 6]. In 2010, the proportion of high school students who consumed SDs and EDs at least once per day was 16% and 5%, respectively [7].

SDs contain carbohydrates, minerals, and electrolytes and are often marketed for the purpose of rehydration [8]. However, drinking water alone provides adequate replenishment in most instances other than prolonged vigorous exercise [8]. Both SDs and EDs have been associated with increased dental erosion, due to their acidity [9] and the presence of citric acid [10]. EDs carry additional consequences due to their stimulatory and performance enhancing effects, derived from ingredients such as caffeine, taurine, guarana, and carbohydrate sweeteners [11, 12]. Unlike caffeinated soda drinks, EDs do not have restrictions on their caffeine concentration nor are they required to have undergone safety testing or placement of appropriate warning labels [6, 13]. As a result, the caffeine content found in EDs can range from 50 mg to 505 mg per can, which can be as much as that found in 14 12-ounce cans of a typical soda [6]. Consumption of EDs may put adolescents at risk of adverse effects of caffeine (elevated heart rate, anxiety, and sleep disturbances) and in one study their consumption was associated with coconsumption of alcohol [6]. A significant proportion of US adolescents regularly consume EDs, ranging from 28% of 12- to 14-year-olds to 34% of 18- to 24-year-olds [13]. The problem extends to other countries, as evidenced by one study in Germany that found 23% of adolescents regularly consuming EDs at <1 can per week and 3% consuming 1 to 7 cans per week [14]. Furthermore, a recent study of US poison center data described the disproportionate exposure of children and young adults to EDs; among cases involving nonalcoholic EDs, over 50% occurred with children under 6 years of age while 68% of cases involving alcoholic EDs occurred with young adults under 20 years of age [15].

Health care providers can play an important role in addressing the issue of SED consumption, through counseling and educating adolescent patients and their families on associated health risks. In 2011, the American Academy of Pediatrics (AAP) recommended routine counseling of children and adolescents for SED consumption; the AAP emphasized that water should be the principle source of hydration rather than SD and that due to the potential health risks of caffeine, any consumption of ED by children should be discouraged [8]. While counseling practices related to SEDs have not been well described, numerous studies have examined counseling practices for general SSB or adolescent obesity. One cross-sectional survey found that 38% of youth participants had discussed SSB with their physicians and 69% of youths reported that they would change their obesity-related behaviors if it were recommended by their physicians [16]. In a separate survey study, youths were more likely to report positive perceptions of a health care provider if they discussed one or more “sensitive health topics,” which included mood, drugs and alcohol, sexuality, and family issues although body weight was not included [17].

A better understanding is needed on how provider characteristics are associated with counseling practices, specifically with regard to SED consumption. The purpose of this study is to examine the prevalence and frequency of SED counseling among health care providers, where counseling is defined as discussions during which patients are advised to limit consumption of SDs and to avoid any consumption of EDs. Furthermore, this study also examines whether provider characteristics are associated with regular SED counseling.

## 2. Methods

Data was obtained from DocStyles 2011, a web-based panel survey developed by Porter Novelli. The survey is designed to provide insight into health care provider attitudes and counseling behaviors regarding a variety of adult and pediatric health issues and to assess their use of health information resources. In addition, the survey includes questions on the provider's height and weight, as well as other questions describing their demographics, health behaviors, and practice characteristics. The Centers for Disease Control and Prevention Human Subjects Review determined that these analyses were exempt from Human Subjects Review because this is a secondary data analysis using data without identifiers.

### 2.1. Survey Participants

This study is based on responses from family/general practitioners (FGPs), internists, pediatricians, and nurse practitioners in DocStyles 2011. For the complete DocStyles 2011 survey, physicians and other health care providers were randomly selected from Epocrates' Honor Panel and Epocrates' Allied Health Panel, respectively. Epocrates' Honors Panel is an opt-in panel of over 190,000 medical practitioners, verified by checking each physician against the American Medical Association's (AMA) master file. Participants for this study were randomly selected through a probability sampling method to meet preestablished quotas for each health care provider type (1,000 primary care physicians, 250 pediatricians, 250 obstetricians/gynecologists, 250 retail pharmacists, 250 nurse practitioners, and 200 registered dietitians) and for the physician sample to match the AMA master file in terms of age, gender, and region. The total sample is not intended to be representative of the national population of health care providers. Participants were paid an honorarium of $40–$50 for completing the survey.

A total of 2,204 health care providers completed the DocStyles 2011 survey. Porter Novelli compared the physician participants, stratified by specialty, to the AMA master file for gender, age, and region of the country and found the average age to be lower compared to national averages. The present study was limited to FGPs (*n* = 544), internists (*n* = 85), pediatricians (*n* = 239), and nurse practitioners (*n* = 178) who regularly see patients ≤17 years old (*n* = 1046). Only these individuals were eligible to respond to the questions regarding counseling of adolescents on SED consumption, per survey protocol. After exclusion of respondents due to ambiguous or inconsistent responses, the final analytic sample was 1014. The authors were unable to assess the comparability of this analytic sample with the AMA master file because the study was restricted to those who see pediatric patients and are of particular specialties. Comparison data were not available for nurse practitioners.

### 2.2. Prevalence and Frequency of SED Counseling

**Figure 1:**
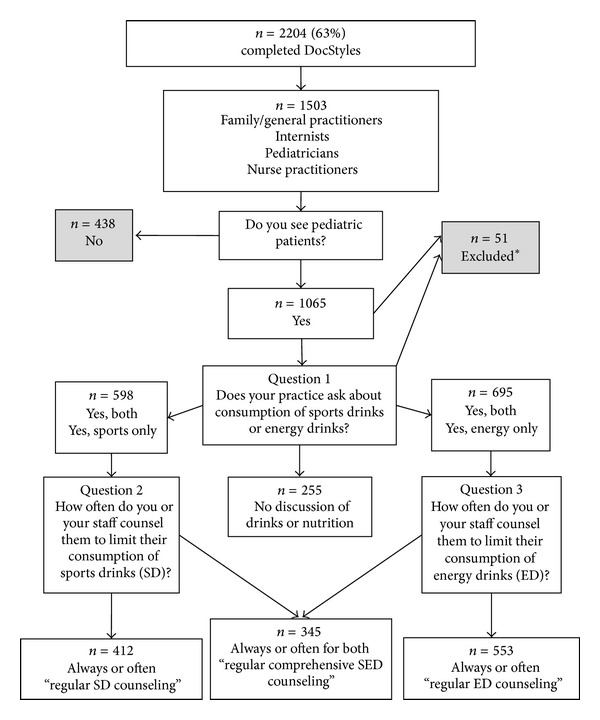
Flowchart of DocStyles 2011 survey questions regarding sports and energy drink counseling practices among healthcare providers. This flowchart depicts the questions used in this analysis of the DocStyles 2011 survey and how providers were categorized regarding their counseling patterns around sports (SD) and energy (ED) drinks. This categorization formed the basis of the analyses. Individuals answering “both” or “SD only” to Question 1 were asked Question 2. Those who responded with “both” or “ED only” were asked Question 3. Regular SD counseling was defined as those individuals who regularly (“Always” or “Often”) counseled on reducing SD consumption. Regular ED counseling was defined similarly. Comprehensive SED counseling was defined as individuals who counseled on “Both drink types” and counseled regularly for both. ∗ indicates patients excluded from sample due to missing/incomplete demographic information (*n* = 10), responses to Questions 2 or 3 that are contradictory to responses to Question 1 (*n* = 9), or answering “Not Sure” to Question 1 (*n* = 32).

The analysis of health care provider counseling of adolescents on SED is based on the three questions listed in Figure 1. Question  1 asks about SED counseling for adolescent patients. Per survey protocol, individuals who responded with “Both drink types” or “Sports drinks only” were asked Question  2, which inquires about frequency of counseling to limit SD consumption. Individuals who responded with “Both drink types” or “Energy drinks only” were asked Question  3, which inquires about frequency of counseling to not drink EDs. The responses were dichotomized in three different ways. Regular SD counseling was defined as those individuals who regularly (“Always” or “Often”) counseled on reducing SD consumption with the comparison group being those who counseled less frequently (“Sometimes,” “Rarely,” or “Never”) or did not provide any counseling. Regular ED counseling was defined similarly. Lastly, regular comprehensive SED counseling was defined as individuals who counseled on “Both drink types” and counseled regularly for both; the comparison group included everyone who provided counseling less frequently or did not provide counseling. In all three outcomes, individuals who responded with “Not Sure” in Question  1 (*n* = 32) or “Sports drinks/Energy drinks are not typically discussed” in Questions  2 (*n* = 2) and  3 (*n* = 7) were excluded due to ambiguity or inconsistency of answers.

### 2.3. Predictors of SED Counseling

To investigate factors associated with SED counseling, personal and medical practice-related characteristics of each participant were analyzed. Personal characteristics include age (≤45 years versus >45 years), sex, race and ethnicity (non-Hispanic white versus all others), weight status determined by body mass index (BMI) calculated from self-reported height and weight (“Normal/underweight” BMI < 25, “Overweight” 25 ≤ BMI < 30 and “Obese” BMI ≥ 30), number of days per week they ate ≥5 cups of fruit or vegetables henceforth called “high” intake (<4 days versus ≥4 days), and number of days they exercised or kept their heart rate up for 30 minutes (<5 days versus ≥5 days). Medical practice-related characteristics included type of specialty, practice setting (individual versus group versus hospital or clinic), affiliation with a teaching hospital, years of practice (≤10 years versus >10 years), number of total patients per week (≤100 versus >100), and perceived financial status of their patients (“Very Poor to Poor,” “Poor to Low Middle,” “Low Middle to Middle,” “Middle to Upper Middle,” and “Upper Middle to Affluent”).

### 2.4. Statistical Analysis

The overall prevalence among health care providers giving regular counseling to adolescent patients regarding SDs, EDs, or both was assessed. Chi-square analysis was used to examine differences in the prevalence of regular SED counseling among participants with different personal and medical practice-related characteristics. Multivariate logistic regression was conducted to determine characteristics independently associated with the three outcomes of interest: regular SD counseling, regular ED counseling, and regular comprehensive SED counseling. The significance level was *P* < 0.05 and the selection criterion for bivariate inclusion in the multivariate model was *P* < 0.20 [13]. All analyses were conducted using SAS 9.2 statistical software (SAS Institute Inc.).

## 3. Results

Approximately three-fourths of providers were non-Hispanic whites, with a relatively even distribution in sex and age (Table 1). Thirty-seven percent of providers were overweight and 15% were obese, 55% reported high fruit and vegetable intake (at least 5 cups per day, ≥4 days per week), and 29% engaged in 30 minutes or more of physical activity ≥5 days per week. Nearly 60% had been practicing for greater than ten years and approximately half (46%) had teaching privileges. The majority (66%) of the participants worked in a group practice, 57% had five or less physicians in their practice, and 58% saw ≤100 patients per week.

### 3.1. Regular Sports Drink and Energy Drink Counseling

Overall, 41% of participants provided regular (*always *or* often*) SD counseling compared to 55% for ED counseling (Table 1). Approximately 42% of individuals never counseled about SDs and 32% about EDs. Female providers exhibited a higher prevalence of regular counseling, at 47% and 61% for SDs and EDs, respectively, compared to 35% and 49% by male providers. Pediatricians had the highest prevalence of regular SD counseling at 55% compared to internists who had the lowest prevalence at 30%. For regular ED counseling, nurse practitioners had the highest prevalence at 65% compared to internists at 43%.

Multivariate modeling found that regular SD counseling was independently associated with being female (adjusted odds ratio (aOR): 1.41 [95% confidence interval (95% CI): 1.07–1.88]), high fruit and vegetable intake (aOR: 1.63 [95% CI: 1.24–2.13]), type of provider, and group practices compared to individual practices (aOR: 0.66 [95% CI: 0.47–0.93]) (Table 2). In the model, compared to pediatricians as the reference group, FGPs (aOR: 0.46 [95% CI: 0.33–0.64]), internists (aOR: 0.33 [95% CI: 0.18–0.59]), and nurse practitioners (aOR: 0.55 [95% CI: 0.36–0.83]) all had significantly lower odds of providing regular SD counseling.

Similar to SD counseling, multivariate modeling found that regular ED counseling was independently associated with being female (aOR: 1.40 [95% CI: 1.06–1.84]), high fruit and vegetable intake (aOR: 1.68 [95% CI: 1.30–2.16]), internists as compared to pediatricians (aOR: 0.56 [95% CI: 0.33–0.96]), and group practices compared to individual practices (aOR: 0.68 [95% CI: 0.48–0.95]) (Table 2). Other provider types did not significantly differ from pediatricians in the adjusted model.

With regard to either regular SD counseling or ED counseling, no statistically significant differences were found with regard to race/ethnicity, BMI, years of practice, teaching privileges, number of physicians per practice, number of patients seen per week, or the provider's perception of their patient population's socioeconomic level (Table 2).

### 3.2. Regular Comprehensive SED Counseling

The third outcome of this study is regular comprehensive SED counseling, which had an overall prevalence of 34% (Table 1). The prevalence of counseling was higher in female providers at 41% compared to male providers at 28%. Among the types of health care providers, pediatricians and nurse practitioners had the highest prevalence of comprehensive SED counseling at 43% and 42%, respectively, compared to FGPs at 29% and internists at 23%. Multivariate modeling found regular comprehensive SED counseling to be independently associated with being female (aOR: 1.44 [95% CI: 1.07–1.93]), high fruit and vegetable intake (aOR: 2.05 [95% CI: 1.54–2.73]), FGPs (aOR: 0.58 [95% CI: 0.41–0.82]) and internists (aOR: 0.37 [95% CI: 0.20–0.70]) compared to pediatricians, and group practices (aOR: 0.59 [95% CI: 0.42–0.84]) compared to individuals practices (Table 2). No other provider characteristics were found to be associated.

## 4. Discussion

We found that one-third of health care providers in this study reported comprehensive SED counseling, indicating that the majority of health care providers who see adolescent patients were not providing both SD and ED counseling regularly. Stratification by provider type found that pediatricians had the highest prevalence of regular comprehensive SED counseling (43%). This is lower than the SSB counseling rate of 65% reported by pediatricians in the 2006 AAP Periodic Survey of Fellows [18]. However, differences may be attributed to the topic counseled upon (SED versus SSB) and how “frequent counseling” was defined by each study. In addition to pediatricians, nurse practitioners also exhibited high counseling rates; these two provider types comprised the two highest prevalences of SD only, ED only, and comprehensive SED counseling. Comparatively, FGPs and internists had significantly lower prevalences of SED counseling.

The finding that pediatricians typically exhibited the highest rates of SED counseling is congruent with previous studies, which have also have found that pediatricians more frequently assessed weight status and provided behavioral counseling than family/general practitioners [19, 20]. Nurse practitioners were more likely to provide SED counseling than FGPs and internists in this study. One possible reason for this difference may be that, in this study, FGPs and internists tend to see fewer pediatric patients per week (FGPs/internists: 23.3 pediatric patient versus nurse practitioners: 29.4 versus pediatricians: 105.0) and may have less experience counseling adolescents. Another reason, though not investigated in this study, is the amount of time nurse practitioners have to spend with each patient and the complexity of cases they manage. Regardless of the cause of the disparities, FGPs and internists likely play significant roles in the health maintenance of adolescent patients. Gaps in counseling practices can be addressed through changes in the training and continuing education of providers. Currently, the differences in provider access and familiarity of AAP counseling recommendations are unknown.

This study also found that the prevalence of regular SD counseling and of regular ED counseling significantly differed overall, at 41% and 55%, respectively. Specifically, this difference varied by provider type, with internists exhibiting the greatest discrepancy (21% higher for ED counseling) in comparison to pediatricians (1% higher for ED counseling). These findings suggest that there are provider-specific differences in how the health risks of these two drink types are perceived and managed.

We found that being female, having high fruit and vegetable intake, being a pediatrician, and operating in an individual practice compared to a group practice were associated with greater odds of providing regular comprehensive SED counseling. Regular SD counseling and regular ED counseling were associated with the same provider characteristics. Some variables identified in previous studies [21, 22] to be related to physician counseling behaviors such as race/ethnicity and BMI were not found to be associated in this study. Compared to Bleich et al., this study contained a similar proportion of overweight/obese providers at 52%, with the difference being a focus on adolescent patients with whom providers may be more motivated to counsel on obesity-related topics.

Given the relatively low rates of regular SED counseling, increasing provider awareness of the health effects of SED is the first step in addressing this issue. Secondly, studies have found that various factors affect how inclined and confident a clinician is in terms of providing health behavior counseling [23, 24]. One study found that physicians with normal BMIs were more likely to discuss weight loss and were more confident in offering diet and exercise suggestions [21]. Another study found that clinicians who personally struggle with making healthy choices, such as avoiding calorically dense foods and beverages, may find it more difficult to counsel patients on that topic [23]. Our data supports that hypothesis, with providers of high fruit and vegetable intake having higher prevalence of SED counseling. Encouraging health care providers to lead healthy lives may contribute to higher prevalences of SED counseling. Ideally, providers can be role models for healthy behavior and create a supportive environment in their clinic or hospital setting and in the community-at-large for patients and families [25].

Other studies have found that another barrier to offering effective health counseling is a lack of training or confidence in behavioral counseling [23]. Techniques such as motivational counseling [26, 27], the 5 A's heuristic [28], and multistage models [29] provide frameworks from which effective changes can be encouraged. Investigators have developed and examined curricula for medical students and residents [30–32], comprised of lectures and opportunities to practice counseling techniques that resulted in subjective increases in confidence in providing health behavior counseling. Lastly, physicians can also learn from effective community-based interventions and adapt them for their patients. For example, physicians can consider explaining caloric information to adolescent patients in terms of physical activity equivalents, a promising intervention used for youth in Baltimore [33].

This study has a number of limitations. First, the sample was drawn from an opt-in database, which is subject to selection bias and may not be representative of health care providers nationally. Second, the responses regarding counseling behaviors are subjective and susceptible to recall bias; thus, reported prevalence may not reflect actual practices. Third, there is limited evidence for or against the assignment of the “Sometimes” response to the “NOT regular counseling” group; however, the data was also analyzed with “Sometimes” assigned to the “regular counseling” group and the results were not significantly different. Finally, the survey did not assess barriers that prevented SD and/or ED counseling from being offered.

With the prevalence of regular comprehensive SED counseling at 34%, increased efforts must be applied to educate adolescent patients and not overlook the associated health risks of high consumption of these beverages. Additional research is required to assess physician opinions and barriers that stand in the way of providing regular counseling. Research and training can help teach health care providers of various disciplines pertinent information and effective counseling techniques for patients and parents.

## Figures and Tables

**Table 1:** Number and proportion (%) of health care providers who responded with always/often counseling (A), sometimes counseling (S), and rarely/never/no counseling (N) of adolescent patients regarding consumption of sports drinks (SD), energy drinks (ED), or both types of drinks (comprehensive SED), by provider characteristics (United States, DocStyles, 2011).

Provider characteristics	Total		Sports drink			Energy drink			Comprehensive SED		
	*N*	%	%A	%S	%N	%A	%S	%N	%A	%S	%N
Overall	1014	100	41	14	45	55	12	34	34	14	52
Sex											
Male	537	53	35*	**15**	**50**	49*	**13**	**38**	28*	**15**	**57**
Female	477	47	47*	**13**	**41**	61*	**9**	**30**	41*	**12**	**47**
Age											
≤45	562	55	40	15	45	55	11	34	34	13	52
>45	452	45	41	13	46	54	13	33	34	14	53
Race/ethnicity											
Non-Hispanic white	726	72	41	14	46	56	12	33	34	14	52
All others	288	28	40	15	45	52	11	37	33	13	55
Body mass index											
Normal/under	489	48	41	15	44	55	13	32	35	13	51
Overweight	377	37	39	13	48	53	10	37	31	12	56
Obese	148	15	44	14	43	57	11	31	37	16	47
Fruit/vegetable intake per week											
<4 days	452	45	33*	**13**	**53**	47*	**13**	**39**	25*	**14**	**62**
≥4 days	562	55	47*	**14**	**39**	60*	**10**	**30**	42*	**13**	**45**
Exercise per week											
<5 days	724	71	39	14	48	53	10	36	33	12	55
≥5 days	290	29	46	14	40	57	14	29	38	16	46
Type of provider											
Pediatrician	234	23	55*	**15**	**31**	56*	**15**	**29**	43*	**13**	**44**
Family/general Practitioner	531	52	35*	**13**	**52**	52*	**10**	**38**	29*	**13**	**58**
Internist	77	8	30*	**16**	**55**	43*	**16**	**42**	23*	**17**	**60**
Nurse practitioner	172	17	44*	**15**	**41**	65*	**11**	**24**	42*	**15**	**42**
Work setting											
Individual	195	19	47	10	43	62*	**7**	**31**	42*	**8**	**50**
Group	665	66	39	14	47	52*	**12**	**36**	31*	**15**	**54**
Hospital/clinic	154	15	40	17	43	56*	**17**	**27**	36*	**16**	**47**
Teaching privileges											
No	550	54	40	13	48	53	10	36	33	13	54
Yes	464	46	42	15	43	56	13	31	36	14	50
Years of practice											
≤10	421	42	39	17	43	56	11	33	34	15	51
>10	593	58	42	11	47	54	12	34	34	12	53
Patients per week											
≤100	585	58	41	14	45	54	11	35	34	14	52
>100	429	42	40	13	46	55	12	33	34	13	53
SES^‡^ of patients											
Very poor-poor	50	5	44	4	52	64	12	24	42	6	52
Poor-low middle	176	17	41	14	45	56	11	33	31	14	55
Low middle-middle	394	39	40	13	47	55	10	35	34	14	52
Middle-upper middle	354	35	41	15	44	53	13	34	34	14	52
Upper middle-affluent	40	4	38	23	40	48	15	38	33	15	53

^‡^“SES of patients” refers to health care providers' perceptions of the socioeconomic status of their patient population; the categories refer to possible responses on the survey without additional clarification.

*Bolded values indicate statistically significant differences due to provider characteristic, with *P* value <0.05 in chi-square analysis.

Percentages subject to rounding error, with sum of row % between 99 and 101%.

**Table 2:** Unadjusted odds ratio (OR), adjusted odds ratio (aOR)^a^, and 95% confidence intervals (95% CI) of providing regular^b^ sports drink (SD) counseling, regular^b^ energy drink (ED) counseling, and regular comprehensive sports and energy drink (SED) counseling^c^ by health care provider characteristics—United States, DocStyles, 2011.

Provider characteristics	Sports drink				Energy drink				Comprehensive SED			
	OR	95% CI	aOR^d ^	95% CI	OR	95% CI	aOR^e^	95% CI	OR	95% CI	aOR^d^	95% CI
Sex												
Male	Ref	Ref	Ref	Ref	Ref	Ref	Ref	Ref	Ref	Ref	Ref	Ref
Female	1.62	1.26–2.08	1.41*	**1.07–1.88**	1.62	1.26–2.08	1.40*	**1.06–1.84**	1.75	1.35–2.28	1.44*	**1.07–1.93**
Fruit/vegetable intake per week												
<4 days	Ref	Ref	Ref	Ref	Ref	Ref	Ref	Ref	Ref	Ref	Ref	Ref
≥4 days	1.76	1.36–2.27	1.63*	**1.24–2.13**	1.72	1.34–2.21	1.68*	**1.30–2.16**	2.15	1.64–2.82	2.05*	**1.54–2.73**
Type of provider												
Pediatrician	Ref	Ref	Ref	Ref	Ref	Ref	Ref	Ref	Ref	Ref	Ref	Ref
Family/general Practitioner	0.44	0.32–0.61	0.46*	**0.33–0.64**	0.84	0.62–1.15	NS		0.55	0.40–0.75	0.58*	**0.41–0.82**
Internist	0.35	0.20–0.61	0.33*	**0.18–0.59**	0.58	0.34–0.98	0.56*	**0.33–0.96**	0.41	0.23–0.74	0.37*	**0.20–0.70**
Nurse practitioner	0.66	0.44–0.98	0.55*	**0.36–0.83**	1.41	0.94–2.11	NS		0.99	0.66–1.47	NS	
Work setting												
Individual	Ref	Ref	Ref	Ref	Ref	Ref	Ref	Ref	Ref	Ref	Ref	Ref
Group	0.71	0.51–0.98	0.66*	**0.47–0.93**	0.68	0.49–0.95	0.68*	**0.48–0.95**	0.62	0.45–0.87	0.59*	**0.42–0.84**
Hospital/clinic	0.75	0.49–1.16	NS		0.79	0.51–1.22	NS		0.79	0.51–1.22	NS	
Age	NS		NS		NS		NS		NS		NS	
Race/ethnicity	NS		NS		NS		NS		NS		NS	
Body mass index	NS		NS		NS		NS		NS		NS	
Exercise per week	NS		NS		NS		NS		NS		NS	
Teaching privileges	NS		NS		NS		NS		NS		NS	
Years of practice	NS		NS		NS		NS		NS		NS	
Patients per week	NS		NS		NS		NS		NS		NS	
SES^‡^ of patients	NS		NS		NS		NS		NS		NS	

^‡^“SES of patients” refers to health care providers' perceptions of the socioeconomic status of their patient population; the categories refer to possible responses on the survey without additional clarification.

^a^Model adjusted for characteristics that met the inclusion criteria of *P* value <0.2 in bivariate analysis.

^b^Regular is defined by responses of *always* or *often* on Question 2 and Question 3.

^c^Regular comprehensive SED counseling defined as providing counseling at a frequency of *always* or *often* for both sports and energy drinks.

^d^aOR for sports drink and comprehensive SED counseling adjusted for sex, fruit/vegetable intake, exercise, provider type, and work setting.

^e^aOR for energy drink counseling adjusted for sex, fruit/vegetable intake, provider type, and work setting.

Ref is reference level for each variable.

Referent categories: male, age ≤ 45, non-Hispanic white, normal/underweight BMI, fruit/vegetable intake <4 days per week, exercise <5 days per week, pediatrician, individual practice, ≤10 years of practice, ≤5 physicians in practice, ≤100 patients per week, and very poor-poor SES of patients.

*Bolded aOR values indicate that 95% confidence intervals do not include 1.

NS indicates nonsignificance of provider characteristic with regard to association with SD, ED, or SED counseling.

## Abbreviations

SSB: Sugar-sweetened beverages
SD: Sports drinks
ED: Energy drinks
SED: Sports and energy drinks
AAP: American Academy of Pediatrics
FGP: Family or general practitioner
AMA: American Medical Association
BMI: Body mass index
aOR: Adjusted odds ratio
OR: Odds ratio.
